# The Effectiveness of Nonprescription Drug Labels in the United States: Insights from Recent Research and Opportunities for the Future

**DOI:** 10.3390/pharmacy6040119

**Published:** 2018-10-26

**Authors:** Jesse R. Catlin, Eric P. Brass

**Affiliations:** 1 College of Business Administration, California State University, Sacramento, CA 95819, USA; 2 Department of Medicine, University of California, Los Angeles, CA 90095, USA; ebrass@ucla.edu

**Keywords:** Drug Facts Label, over-the-counter drugs, label comprehension, self-selection, actual use, pharmacists, technology, consumer

## Abstract

Despite providing a consistent and comprehensible format for over-the-counter (OTC) drug communication, research suggests important limitations in the communication effectiveness of the Drug Facts Label required on OTC drugs in the United States. This literature is reviewed and some of these critical limitations of the Drug Facts Label (DFL) highlighted. These include difficulty communicating complex information that requires integration of multiple pieces of label information and limited adaptability to serve the unique needs of individual populations (e.g., low literacy or older consumers). Potential ways to improve the DFL’s communication effectiveness are identified along with complementary opportunities to improve OTC drug communication by leveraging the role of pharmacists and use of adjunctive technologies.

## 1. Introduction

Self-care of minor health problems is facilitated by the availability of a wide range of nonprescription, or over-the-counter (OTC), drugs. The United States regulatory framework ensures that OTC drugs are safe and effective when used as directed. OTC drug labels serve as the primary means of communicating drug-specific information to consumers. By definition, OTC drugs are available to consumers without a requirement for consulting with a healthcare professional. As a result, an OTC drug’s label must on its own be able to effectively communicate to an average consumer the information required for safe and appropriate usage. In the United States the required Drug Facts Label (DFL) was designed to enhance readability and accessibility of information while ensuring a more consistent label format across OTC products [1]. Initial tests by the United States Food and Drug Administration (FDA) found that the DFL consistently outperformed previously used labeling in terms of consumer preference and readability [1]. Follow-up studies also found that the DFL facilitated faster acquisition of information compared to prior labeling [2]. However, comprehensive testing to optimize the DFL format was not done, nor was full testing done to assess whether the format was optimal for all potential OTC products.

The utility of the DFL is evidenced by the wide use of OTC drugs by consumers to meet a variety of health needs with an overall outstanding safety record. However, the DFL by nature is a static, non-customizable, text-based communication tool and research has suggested that expanding the scope of indications and drugs available to consumers OTC may require changes to the DFL. The purpose of this review is to provide an overview of research on OTC labeling during the nearly 20 years since the introduction of the DFL. In doing so, the DFL’s effectiveness as a communication tool is assessed and opportunities for improvement in consumer-directed OTC communications are highlighted, emphasizing potential avenues to leverage pharmacists’ expertise and adjunctive technologies.

## 2. The Drug Facts Label as a Communication Tool

While an important step forward in providing a more systematized and consumer-focused method for communicating OTC drug information, the question remains as to whether the DFL is an optimized communication tool. In its pivotal role, the DFL faces a myriad of challenges and must serve numerous communication objectives. The DFL must effectively communicate a number of specific messages required for the safe and effective use of the drug. Conceptually, these messages may be divided into those required for self-selection, appropriate use of the drug, and deselection if required (Table 1). Self-selection refers to the assessment of whether the drug is “right for me” based on the consumer’s specific health status. Appropriate use includes information on dose and duration of use. Deselection, or discontinuation of use, may be necessary if adverse events develop or the consumer’s health status changes. Each message must be structured in language and format so that the average consumer can understand the material. For some products, a dozen or more distinct messages may be required, posing significant challenges for manufacturers to create effective labels and for consumers to access and heed the information.

A well-designed DFL will be ineffective if not read by consumers. Unfortunately, multiple studies suggest that less than half of consumers read the entire package labeling before taking OTC medicine [3,4]. For example, only 48% of subjects stated that they always read the usage instructions for OTC pain relievers before use [3]. Similarly, another study found that only 42% of subjects said they read everything on the label when taking an OTC medication for the first time and only 26% report reading the active ingredients at first use [4]. Eye-tracking studies also suggest consumers spend less time viewing warnings compared to other aspects of package labeling (e.g., the brand name) [5,6]. Indeed, brand names may bias some consumers against comprehensive review of the DFL [7].

Further, even when consumers read and understand the information on the DFL, the question remains as to whether they behave consistently with the information provided. Unintentional harm from improper use of OTC products is an important public health concern. Research shows that many consumers exceed daily dosage recommendations for products such as acetaminophen [8,9] and nonsteroidal anti-inflammatory drugs (NSAIDs) like ibuprofen and naproxen [4,10].

These observations make clear the complexity of DFL construction and its importance for safe and effective use of OTC drugs. The challenges faced by consumers in using the DFL also make clear the importance of the pharmacist and emerging technologies as potential resources for consumers making OTC product decisions, and necessitate that pharmacists understand the limitations of the OTC DFL.

## 3. Label Comprehension, Self-Selection, and Actual Use Studies

Studies of the DFL’s ability to achieve central communication objectives for specific drugs are often conducted as part of an application to switch a drug from prescription to OTC status. Such studies are used by regulators to determine whether consumers will be able to safely and effectively utilize a drug in an OTC environment (i.e., using information on the proposed labeling instead of a healthcare professional) [11]. Some consumer research studies of the DFLs for specific drugs are published in peer-reviewed journal articles [12,13,14,15,16,17], while others can be found in the meeting materials posted by the FDA’s Nonprescription Drug Advisory Committee [18].

OTC label studies include label comprehension studies, self-selection studies, and actual use studies [19]. Label comprehension studies evaluate consumers’ ability to read and comprehend the DFL. In a typical label comprehension study, consumers are provided the proposed DFL and their understanding of label information and/or behavioral intentions are measured [20,21]. A label comprehension study is not a memory test, and subjects have access to the DFL while answering the questions. A typical self-selection study investigates consumers’ ability to apply DFL to their own personal medical situation and determine whether the drug is appropriate for them to use [22]. Actual use studies assess consumer decision-making and behaviors when using the drug in as naturalistic a setting as possible. An actual use study evaluates the DFL by attempting to simulate actual purchase environments in which participants make self-selection decisions (i.e., deciding whether to purchase and use a particular drug), whether they deselect for continued use based on their response to the drug, and if they follow the directions for use [19]. Diaries or follow-up surveys are often conducted with purchasers of the drug to evaluate their actual use of the product (e.g., dosage and frequency, etc.) [14,15,17,23]. In some situations, a self-selection study may be conducted as a component of an actual use study [22].

Importantly, the results of label comprehension studies, self-selection studies, and actual use studies may need to be viewed as idealized estimates of DFL comprehension and appropriate usage in the post-approval marketplace. Label comprehension and self-selection studies allow full access to the DFL while answering questions and thus inherently include some degree of cuing to specific label information. Similarly, despite efforts to maintain realism in the design of self-selection and actual use studies, the mere fact that an individual knows they are participating in a study (e.g., completing a questionnaire or filling out a diary each day while using a medication) may bias behaviors towards DFL compliance. These issues are explored further in the sections that follow.

### 3.1. Label Comprehension

Results of label comprehension studies often suggest high levels of comprehension when testing understanding of individual statements or warnings on the label. For example, in a label comprehension study for omeprazole, results suggested that 95% of subjects understood that the dose was 1 tablet, 91% understood the product should be taken for 14 consecutive days, and to contact a health care professional before using beyond 14 days [24]. For lovastatin, a label comprehension study showed comprehension rates above 90% on questions measuring understanding of specific drug information such as the condition treated by the drug, frequency of use, and dosage [25]. Label comprehension studies for lovastatin also showed similarly high rates (~90%) of comprehension for warnings related to individual safety factors such as age, pregnancy, breast feeding, liver disease, and the need to discontinue use if one develops muscle pain [26].

### 3.2. Self-Selection and Actual Use

Results assessing intended or actual behaviors such as self-selection in a self-selection study or product use in an actual use study may exhibit lower rates of compliance/heeding of the label than suggested by label comprehension results. In contrast to label comprehension study measures of individual label statements, proper self-selection and use can be more complex, requiring integration of multiple pieces of information (e.g., simultaneously meeting multiple criteria for appropriate use or adhering to the correct dosage amount, interval, and duration of use). For example, an actual use study of omeprazole found that 19% of subjects did not meet all six criteria specified on the label for appropriate self-selection, 21% did not comply with the recommended drug regimen, and 5% exceeded the recommended number of doses [27]. FDA reviewers of an actual use study for lovastatin determined that only 1% (*n* = 3 out of 209) of subjects who believed they could immediately start taking the drug were correct on all of the label criteria specified for appropriate self-selection, including age, low density lipoprotein cholesterol concentration, risk factors, medical history, pregnancy status, and other medication usage [26]. Of course, expecting consumers to answer correctly across multiple different items presents a significant challenge for any means of communication. Nevertheless, these examples highlight the difficulty in communicating complex information using the DFL alone and the room for improvement that could be facilitated by alternative labeling strategies or by supplemental assistance from pharmacists or adjunctive technologies.

It is also critical to interpret wrong answers in label comprehension studies, incorrect self-selection in self-selection studies, and/or inappropriate usage behavior in actual use studies in the context of the clinical importance of the message being assessed [28]. In most cases, small deviations from the desired behavior do not affect either efficacy or safety of the drug, and can thus be accepted. However, for messages where noncompliance poses a serious risk to the consumer a high magnitude of correct comprehension/behavior should reasonably be required.

Overall, these results suggest that often a DFL can be developed which performs reasonably well in label comprehension study settings where individual pieces of label information are assessed. However, the results of these self-selection studies and actual use studies suggest that the DFL struggles to communicate more complex or multifaceted concepts required to make decisions about self-selection and use. Furthermore, despite the large numbers of label comprehension, self-selection, and actual use studies conducted and their role in regulatory decision-making, their ability to predict real-world behaviors is uncertain. Studies of drugs already approved for OTC use suggest instances of consumer misconceptions of label elements or noncompliance. For example, a recent study found that only about 24% of users of acetaminophen-containing products knew both that acetaminophen was an active ingredient and that they should avoid concomitant use of more than one product containing acetaminophen [29]. Follow-up diaries showed that subjects who lacked knowledge about active ingredient contents and/or concomitant use were more likely to have deviated from the label directions when taking drugs containing acetaminophen [29]. In another example, Shi et al. (2004) found that 54% of purchasers of histamine2-receptor antagonists made self-selection errors (i.e., chose the product when they did not meet the criteria listed on the label). Further, a study of female purchasers of OTC phenazopyridine found significant gaps in knowledge about the drug, including the indications for use of the drug and its function (e.g., 43% incorrectly believed phenazopyridine functions as an antibiotic as opposed to its actual function as a urinary tract analgesic) [30].

The overall limitations of the DFL as assessed through label comprehension, self-selection, and actual use studies has led to more focused research to identify individual-level factors associated with a failure to comprehend and heed DFL instructions. An understanding of these factors is also an important step in improving DFL effectiveness and leveraging the role of the pharmacist and technology tools.

## 4. Individual-Level Factors and the DFL

### 4.1. Health Literacy, Visual Ability, and Language Barriers

Health literacy has been defined as “the degree to which individuals have the capacity to obtain, process, and understand basic health information and services needed to make appropriate health decisions” [31]. Though some have raised concerns about construct validity and the existence of several different measurement tools [32,33,34], health literacy is a major consideration in DFL studies. Indeed, many label comprehension, self-selection, and actual use studies report outcomes separately by literacy level in an effort to address concerns that some groups may not be as well-equipped to process DFL information. For example, studies have found that limited health literacy is associated with less knowledge about OTC active ingredients and is an impediment to label comprehension [3]. Additionally, a study found that individuals with limited health literacy were less likely to consider active ingredient information when making decisions about pediatric cough and cold medication [35]. Similarly, consumers with limited health literacy were more likely to exceed recommended dosages by taking two or more acetaminophen-containing products simultaneously in a simulated usage scenario [36].

Limited health literacy and visual impairment were both found to be independently associated with improper OTC dosing [37]. Having English as a second language is also associated with lower health literacy (including when measurement instruments are translated into an individual’s native language) [38,39], although language barriers can independently impede label communication [40]. In addition, a quantitative analysis of label language complexity found that the reading ease and grade level scores for a sample of 40 OTC products were beyond the capabilities of an average consumer [41]. Adding a further challenge, FDA guidelines specify use of 6 point font for the body text of the DFL [42] which may impede communication with visually impaired individuals or older adults [43].

To summarize, the DFL is a static, text-based medium written in English, which means that it is inherently limited in its ability to serve the needs of limited literacy, visually impaired, and/or non-English speaking populations. These populations are thus vulnerable to poor comprehension and heeding of the DFL.

### 4.2. Older Adults

Older adults have been identified as an important group for study with respect to OTC use as they represent a growing segment of the population and are heavy users of OTC (and prescription) products [38,44]. One study found that younger consumers processed label information faster than older consumers, although label comprehension did not differ across groups and was relatively high overall [45]. Importantly, as a group, older adults (age 65 and up) exhibit lower levels of health literacy than younger age groups [44] which, as discussed above, inhibits understanding of and compliance with DFL instructions [46]. Potentially offsetting these factors, older consumers were found to be more engaged when making OTC purchases and more likely to read the label completely than younger consumers [47]. Studies have found no age-related differences in the likelihood of exceeding the dosage of acetaminophen [8] or NSAIDs [10], perhaps implying that increased engagement or label reading by older consumers may compensate for other disadvantages. Nevertheless, while age may heighten the level of personal relevance or involvement with OTC drug purchases, there are issues inherent to aging (e.g., decreased cognitive function, reduced vision) that may hinder effective interaction with OTC drug labeling. For instance, older adults preferred and performed better in label comprehension tests when the labels used larger print sizes (e.g., 10 and 7 point font vs. 4 point font) [43].

### 4.3. Knowledge, Attitudes, and Beliefs

Preexisting attitudes and beliefs about OTC drugs can limit attention to and/or result in overriding of DFL instructions. For instance, individuals perceive OTC drugs as less risky than prescription drugs [48] and consumers may hold the erroneous belief that OTC drugs are essentially risk-free even if taken incorrectly [49]. Likewise, some individuals believe that they can choose their own dose regardless of DFL instructions and this belief is associated with exceeding dosage limits for common OTC products [8,10]. Further, 68% of participants in a study of OTC histamine2-receptor antagonists stated that the package labeling had little to no influence on their decision-making [50]. In another study, 51% of participants stated they would be unlikely to read allergy-related information on an OTC label and 80% stated that they would be unlikely to keep the medication packaging (carton) for reference when they begin using the drug [51]. Therefore, even if consumers attend to and understand the information provided in the DFL, their existing beliefs and attitudes may lead them to behave discordantly or to ignore the package labeling altogether. Such misconceptions and apathy toward OTC instructions suggests a broader need to educate consumers about OTC products in a way that goes beyond what the DFL can be expected to accomplish on its own. As credible experts located at the point-of-purchase, pharmacists are well-suited to deliver this type of information.

## 5. Tests of Modifications to the Drug Facts Labeling

In the context of the limitations identified above, a variety of studies have investigated modifications to the DFL, such as additional or revised warnings and graphical icons, to improve communication of specific drug information.

### 5.1. Warnings

As noted above, consumers’ preexisting beliefs about the safety of OTC medications may result in their discounting of label warnings [49] and thus efforts have been made to increase the effectiveness of these warnings. Acetaminophen is a safe OTC analgesic when used as directed, but can cause liver injury when label doses are exceeded. A label comprehension study of revised liver damage warning for acetaminophen found that labels more clearly stating organ damage along with more prominent information about the seriousness of overdose and how to take action led to more accurate behavioral intentions than with existing OTC labeling [52]. Moreover, a within-subjects study found that perceived risk of liver damage for acetaminophen was higher when labels contained revised warnings from the FDA (“organ-specific warning label”) [53]. Studies also suggest that details matter when it comes to warning design and highlight the importance of consumer testing. A test of several alcohol warnings on acetaminophen labeling found that recall was highest for an explicit warning featuring red print and detailing the hazards of combining acetaminophen with alcohol [54]. In addition, consumers in a study of OTC ibuprofen labeling preferred more explicit warnings using bulleted, plain language to communicate drug allergy information [55]. These results from simulated settings highlight the potential utility of warnings, but also emphasize the importance of validation using rigorous consumer-testing. Unfortunately, the addition of warnings to the DFL is frequently recommended without such testing [56,57,58]. As a result, there is little data to validate the effectiveness of such warnings nor the consideration that continued addition of warnings adds to the overall length and complexity of the DFL for some products. Highlighting one warning may focus a consumer’s attention to that specific warning but result in less attention to other portions of the label. This represents yet another area where pharmacists and technology can complement the DFL to help consumers determine which warnings are most relevant and heed them accordingly.

### 5.2. Icons

Icons provide an alternative to text for communicating information. Icons have been studied in an effort to enhance awareness of acetaminophen as the active ingredient as a means to reduce the risk of overdose (e.g., by concomitant use of two medications containing acetaminophen). In a qualitative focus group and interview study, consumers supported the idea of including icons on OTC packaging to help facilitate identification of acetaminophen as the active ingredient and they also responded favorably when shown prototype icons for this purpose [3]. In a comparative study of prototype acetaminophen icons, participants preferred icons that could be more directly connected to the active ingredient, such as using icons with the letters “Ac”, “Ace”, and “Acm” instead of “APAP” (an often used abbreviation for the name of the chemical compound) [59]. A study involving hypothetical scenarios found that an acetaminophen icon (vs. no icon) reduced dosing errors due to concomitant use of two medications containing acetaminophen [60]. Of note, this study also suggested that the effect of the icon was larger among individuals with limited literacy (less than 9^th^ grade level) suggesting the potential utility of using graphical or visual cues to more effectively communicate with this segment of consumers. An experimental study of parents found that pictographic icons that graphically illustrated proper dosing may help limited literacy individuals avoid dosing errors for liquid preparations of pediatric acetaminophen [61]. However, it is important that consumers understand what a particular icon means through prior orientation and education efforts [49].

In general, evidence supports the potential for enhanced warnings and icons to aid in communication effectiveness of specific drug information. However, the design details are important and icons must be carefully designed and tested to ensure the best chance of success [49,54]. Again, it is important to note that the studies to date have taken place in experimental or hypothetical settings and it is still unknown whether their effects will translate to the general marketplace. Another consideration that has yet to be explored systematically is how modifications to certain aspects of the labeling (e.g., icons or warnings) impact attention to or comprehension of other aspects of the labeling. A strategy of continuously adding more content to the label (icons, warnings, etc.) increases the likelihood of information overload [62] and makes it more difficult for any individual consumer to extract the information most relevant to them. This reinforces the appeal of dynamic, customizable resources such as pharmacist and technology-based communication which can be tailored to the individual circumstances of each consumer.

## 6. Discussion and Future Opportunities

Despite the discussed limitations in the communication effectiveness of the DFL, current OTC drugs maintain a remarkable safety profile overall and are used by the vast majority of consumers without issue. Yet, it is clear from prior research that the DFL falls short in some areas, most notably in the ability to tailor the communication to an individual user’s specific situation (i.e., other medications, literacy level, language ability, age, pre-existing beliefs/attitudes, etc.) or when more complex decision algorithms are required (e.g., statins). In addition to the improvements in the DFL itself suggested above, expanding the role of pharmacists and leveraging technology offer opportunities to further improve consumer communication and decision-making on the use of OTC drugs (see Table 2).

### 6.1. Expanded Role of Pharmacists

Pharmacists remain a trusted resource [63] located at the point-of-purchase for many OTC drugs. Increasing dialogue between pharmacists and consumers of OTC drugs would bridge some of the previously identified gaps in OTC label communication. Pharmacist-consumer interaction at the point of purchase can allow for pharmacists to ask questions and communicate key information tailored to an individual’s specific situation. For example, pharmacists can provide a more detailed discussion of risks or side effects [64] which may be frequently underestimated by consumers of OTC products [49]. Pharmacists can also help consumers navigate more complex drug information, including decisions involving integration of multiple criteria (e.g., self-selection of a statin). The utility of pharmacist-consumer interaction is even more important as drugs with more complex self-selection algorithms or a potential for serious adverse side effects are considered for prescription-to-OTC switch [65,66]. Further supporting this opportunity, pharmacist advice and guidance for OTC drugs has previously been found to help consumers make more appropriate medication decisions [67].

Consumer-pharmacist interactions can only mitigate the limitations of the DFL if the consumers who need help recognize the need and the availability of pharmacist assistance. This represents another important opportunity for consumer education. Conversely, pharmacist workloads are high in the United States [68] and consumers may be frustrated by delays in being able to talk to a pharmacist. In addition, there are additional barriers related to economic incentives (e.g., lack of payment and/or time spent away from other revenue-generating activities) for OTC drug consultations and some pharmacists may need additional training on how to best navigate OTC-related consumer interactions [69]. Thus, steps should be taken to facilitate these communications in the contemporary pharmacy environment [70]. These steps could range from consumer education campaigns, redesigning the physical layout of pharmacies to promote communication [71], and/or supplemental training for pharmacists. Regulatory agencies and other organizations can also work to incentivize increased consumer-pharmacist interaction related to OTC drugs. In selected cases, interaction between pharmacists and consumers could be encouraged by requiring that a specific OTC drug only be sold at locations and time when a pharmacist is available or in extreme cases that certain drugs be located “behind-the-counter” of the pharmacy. At the same time, it will also be important to design and implement these efforts with an eye toward continuous evaluation and identification of best practices.

### 6.2. Adjunctive Technologies

Despite advances in the use of visual display panels to interact with individuals and the rise in the use of smartphones and other connected devices, OTC labeling and communication efforts have yet to leverage such technological advances. Many consumers are already using tablet devices to provide medical histories when they visit physicians or mobile applications to monitor aspects of a healthy lifestyle. These same technologies can be used as adjuncts to the DFL to aid in consumer communication and decision-making. The FDA has recently acknowledged the potential to harness these capabilities in their July 2018 draft guidance on Innovative Approaches for Nonprescription Drug Products [72]. This draft guidance suggests FDA willingness, for regulatory purposes, to consider adjunctive technologies, such as mobile applications, multimedia displays, websites, etc. as part of the labeling or as conditions of safe use, in addition to the DFL. While a comprehensive review of adjunctive technologies is beyond the scope of this review, broad applications can be easily conceptualized. For instance, interactive decision aids could be implemented at the point-of-purchase that could allow consumers to input personal characteristics or symptoms and receive guidance for self-selection or other pertinent drug information (e.g., taking into account other medications or allergy information) [73]. Technology facilitates multimedia (text, audio, and/or video) delivery of OTC drug information which may be more engaging and persuasive for some users and more accessible to those with limitations related to literacy, vision, or language. Mobile and/or online applications can be developed to communicate with consumers post-purchase in ways that are impossible with current labeling. Post-purchase communications could include reminders or ongoing instructions that are customized to the individual user. Technologies could also be used to measure usage patterns or even adverse events. Of course, rigorously designed studies will be needed to test, validate, and optimize these systems.

## 7. Conclusions

Expanding access to safe and effective drugs without a prescription aligns with the desire of consumers to be more engaged in their own health care and society’s need for efficient health care delivery [74]. Despite its success to date, the inherent limitations in the DFL discussed above represent a potential barrier to optimizing and increasing OTC options for consumers. Recognizing these limitations is an important step in improving OTC drug communication. Leveraging pharmacist expertise and technological capabilities to provide a more dynamic, user-oriented experience have the potential to move beyond what can be done with an optimized, customized DFL. Doing so has the potential to improve consumer outcomes through better comprehension and compliance for existing drugs and could also expand consumer access to additional drugs where the current DFL label alone would be insufficient for OTC approval.

## Figures and Tables

**Table 1:** Examples of key messages in the Drug Facts Label taken from actual OTC products.

Message Purpose	How Is Message Used?	Examples Messages on DFL
Self-selection	Information required to determine if the drug is right for their condition based on their personal health status	**Uses:** “Temporarily relieves minor aches and pains” **Do not use:** “If you ever had an allergic reaction to any other pain reliever” **Ask a doctor before use if:** “The stomach bleeding warning applies to you” **Ask a doctor or pharmacist before use if you are:** “Taking any other drug” **Warnings:** “This product contains an NSAID which may cause severe stomach bleeding. The chances are higher if you are age 60 or older” **If pregnant or breast feeding,** “ask a health professional before use”
De-selection	Alerts consumer of need to discontinue use if inadequate response, a potential adverse event develops, or if there is a meaningful change in their health status	**Stop use and ask a doctor if:** “Pain gets worse or lasts more than 10 days” Information in **Do not use, Ask a doctor before use if, Ask a doctor or pharmacist before use if you are**, **Warning**, and **If pregnant or breast feeding** sections may apply if health status changes
Directions for safe use	Instructs consumer on how to use the product to maximize benefit and minimize risk	**When using this product:** “Take with food or milk if stomach upset occurs” **Directions:** “Take 1 tablet every 4 to 6 h while symptoms persist”

**Table 2:** Challenges to Drug Facts Label (DFL) effectiveness and possible mitigation strategies.

Challenge to DFL Effectiveness	Description(s)	Possible Mitigation Strategies
**Challenge 1:** Static and non-customizable	Cannot uniformly meet the needs of specific consumer populations, for example: Limited literacy Visually impaired Language barriers Older consumers Pre-existing beliefs/attitudes that override DFL messaging	**Alternative DFL Strategies** Icons or enhanced warnings calling attention to critical drug-specific information ○ Must also ensure consumers can understand the meaning of icons (e.g., including text descriptions or through prior orientation/education efforts) Larger text to enhance accessibility of key drug-specific information **Pharmacist** Credible, trusted resource at the point-of-purchase Engage with consumers to understand individual needs/limitations Provide information and guidance most relevant to each consumer Educate consumers about safety/risks associated with OTC drugs **Technology** Dynamic, customizable approaches with flexible implementation ○ Implementable via mobile application, kiosk/display at point-of-purchase, online, etc. Query individual characteristics and deliver personalized information Can use multimedia to create more engaging and/or persuasive content Accessible delivery of information (e.g., text, sound, and/or video)
**Challenge 2:** Communicating complex, multi-attribute criteria for decision-making	Consumers have difficulty understanding more complex DFL information Consumers struggle to integrate multiple DFL criteria necessary for appropriate self-selection and usage decisions	**Alternative DFL Strategies** Icons or enhanced warnings to that clearly highlight most important self-selection criteria May be combined with additional education efforts to assist consumers with interpreting DFL information **Pharmacist** Guide consumers through each aspect of self-selection criteria Dialogue to confirm understanding of usage instructions Opportunity to answer questions and/or provide supplemental information about directions for safe use and de-selection **Technology** Ability to create interactive, step-by-step decision aids Post-purchase follow-up ○ Real-time collection of usage information ○ Reminders, reporting of adverse events, etc.
